# Supplementary material for: Microbial Fe(III) reduction as a potential iron source from Holocene sediments beneath Larsen Ice Shelf
Source: Nat Commun. 2019 Dec 19;10:5786. doi: 10.1038/s41467-019-13741-x (PMC6923428; doi:10.1038/s41467-019-13741-x)
Supplement: Supplementary file 2 — Supplementary Information [file 41467_2019_13741_MOESM2_ESM.pdf]

Supplementary Information

Microbial Fe(III) reduction as a potential iron source from Holocene sediments beneath Larsen Ice Shelf

Jaewoo Jung and Jinwook Kim* et al.

Department of Earth System Sciences, Yonsei University, Seoul 03722, Korea

*Correspondence and requests for materials should be addressed to J.K. (email: jinwook@yonsei.ac.kr)

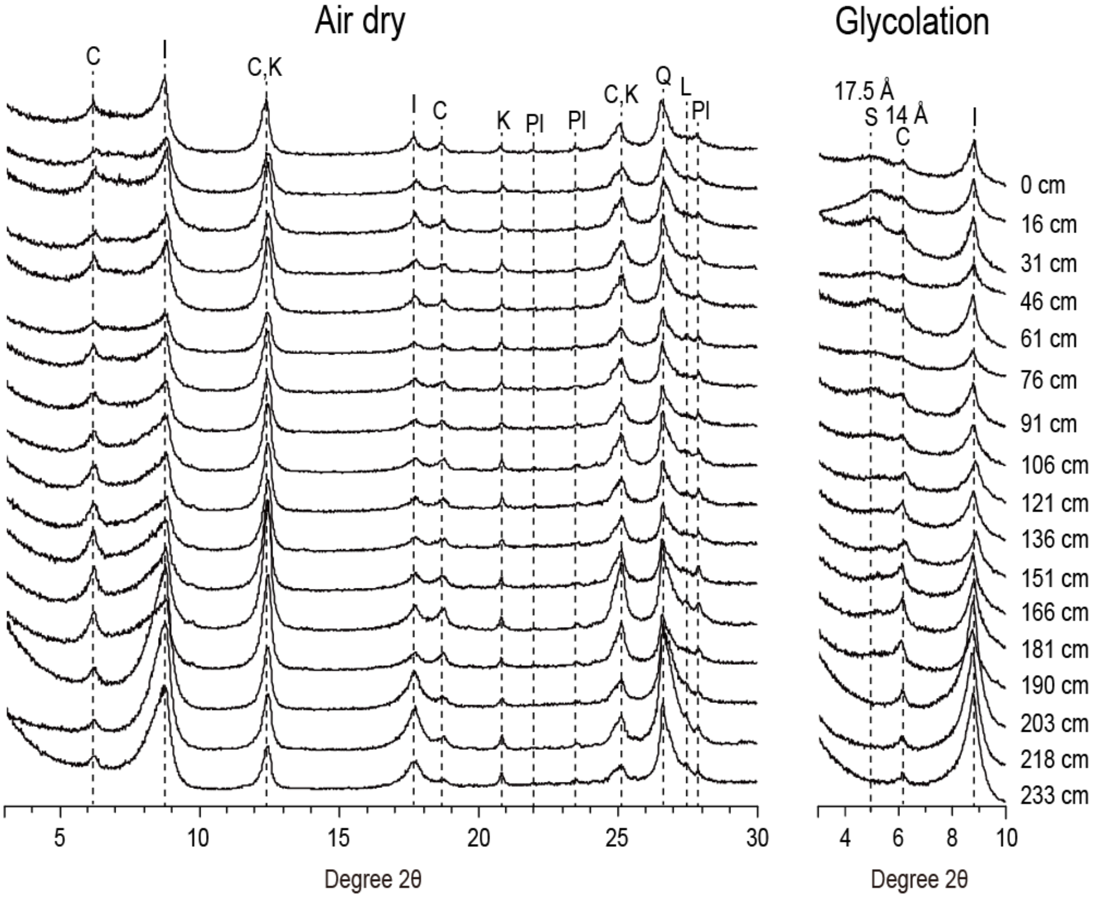

Supplementary Figure 1. X-ray diffraction (XRD) patterns of air-dried and ethylene glycolated clay (<2 μm) in sediment core from site EAP13-GC16B at various depths. There is a clear separation of chlorite (14 Å) and smectite (17.5 Å) for the glycolated samples (S: smectite, C: chlorite, K: kaolinite, I: illite, Pl: plagioclase, Q: quartz, L: lepidocrocite).

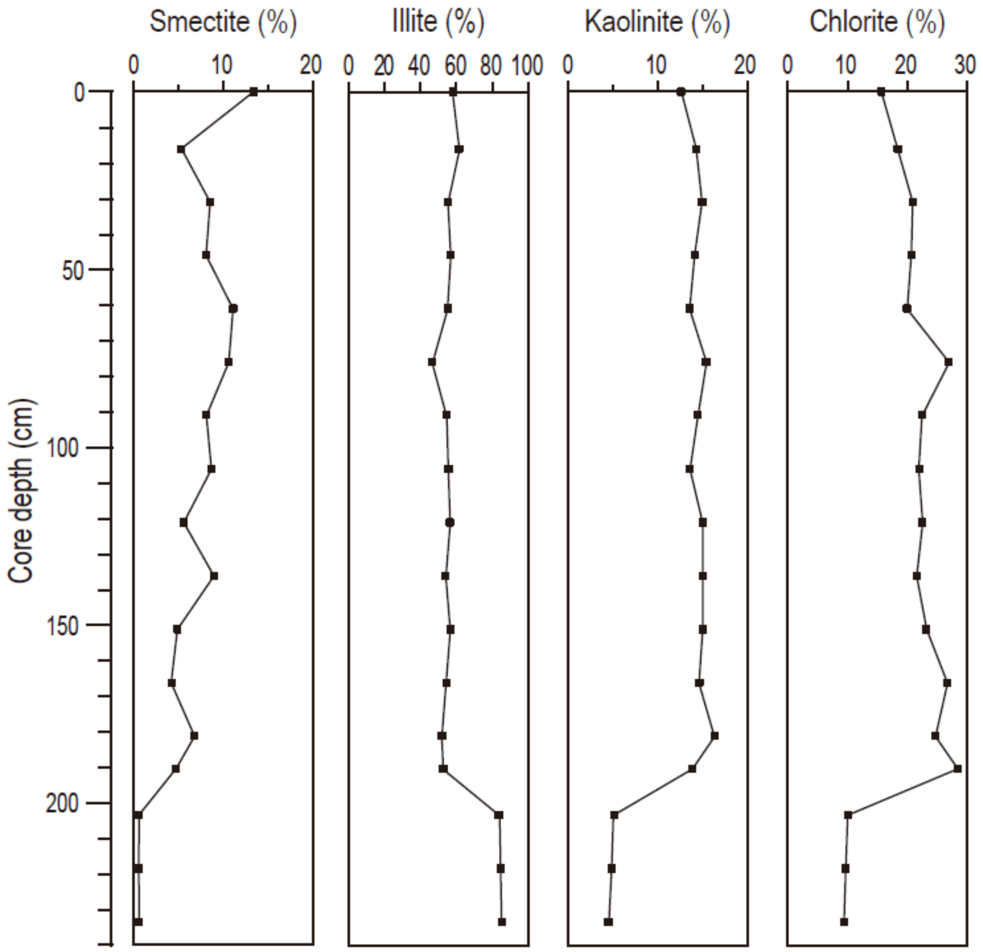

Supplementary Figure 2. Depths profiles of clay minerals in the EAP13-GC16B core. The depth profiles of clay minerals shows that illite is dominant (50-60 %) compared with smectite (~10 %), chlorite (~20 %), and kaolinite (~15 %).

Unit	Depth	$\delta^{13}C$ (VPDB)	$\delta^{15}N$ (AIR)	Unit	Depth	$\delta^{13}C$ (VPDB)	$\delta^{15}N$ (AIR)
U1	0	-25.00	4.39	U2	52	-26.02	2.85
	2	-25.82	4.38		56	-25.97	3.03
	4	-25.93	4.38		62	-26.02	3.26
	6	-25.75	4.48		66	-26.13	3.01
	8	-25.86	3.55		72	-25.91	2.74
	10	-26.08	3.98		76	-26.12	3.45
	12	-25.79	4.17		82	-26.14	3.32
	14	-25.76	3.53		86	-26.02	2.66
	16	-24.60	3.20	U3	92	-26.22	2.21
	18	-25.75	3.58		96	-26.65	1.38
	20	-25.98	2.45		102	-26.58	2.48
U2	22	-25.94	3.44		112	-26.21	2.86
	24	-25.80	3.39		122	-26.39	0.13
	26	-25.73	3.42		132	-26.60	0.25
	28	-25.90	3.36		142	-26.54	2.20
	30	-25.71	3.48		152	-26.52	3.10
	32	-25.77	3.53		162	-26.60	2.34
	34	-24.60	3.22		172	-26.52	1.48
	36	-25.55	3.40		182	-26.54	2.79
	38	-25.72	2.56		192	-26.16	2.48
	42	-26.06	2.77	U4	196	-26.59	-2.87
	46	-26.15	3.53		228	-26.15	-1.18

Supplementary Table 1. Isotopic composition ($\delta^{13}C$ and $\delta^{15}N$) of core EAP13-GC16B. The stable carbon isotope ratio, $\delta^{13}C$, is measured against Vienna Pee Dee Belemnite (VPDB).

Unit	Sample No.	Depth(cm)	IC_air (°Δ2θ)			Average	Standard error	IC_gly (°Δ2θ)			Average	Standard error
			1	2	3			1	2	3		
1	1	0	0.605	0.576	0.458	0.546	0.037	0.411	0.390	0.430	0.410	0.009
	2	16	0.684	0.701	0.769	0.718	0.021	0.448	0.452	0.442	0.447	0.002
2	3	31	0.686	0.728	0.894	0.769	0.052	0.460	0.475	0.460	0.465	0.004
	4	46	0.634	0.694	0.934	0.754	0.075	0.436	0.441	0.427	0.435	0.003
	5	61	0.685	0.662	0.570	0.639	0.029	0.398	0.406	0.392	0.399	0.003
	6	76	0.781	0.887	1.309	0.992	0.132	0.497	0.507	0.483	0.496	0.006
3	7	91	0.936	0.949	1.001	0.962	0.016	0.542	0.535	0.551	0.543	0.004
	8	106	0.764	0.813	1.012	0.863	0.062	0.529	0.540	0.516	0.528	0.006
	9	121	0.951	0.968	1.034	0.984	0.021	0.617	0.640	0.599	0.619	0.010
	10	136	0.981	0.929	0.721	0.877	0.065	0.544	0.550	0.528	0.541	0.005
	11	151	0.892	1.010	1.482	1.128	0.147	0.621	0.631	0.615	0.622	0.004
	12	166	1.070	1.085	1.145	1.100	0.019	0.588	0.589	0.580	0.586	0.002
	13	181	1.076	1.019	0.788	0.961	0.072	0.602	0.602	0.593	0.599	0.002
	14	190	1.094	1.108	1.162	1.121	0.017	0.629	0.630	0.622	0.627	0.002
4	15	203	0.808	0.792	0.728	0.776	0.020	0.550	0.484	0.623	0.552	0.033
	16	218	0.828	0.773	0.553	0.718	0.069	0.542	0.443	0.640	0.542	0.046
	17	233	0.788	0.800	0.848	0.812	0.015	0.546	0.530	0.556	0.544	0.006

Supplementary Table 2. Illite crystallinity values from clay minerals in the <2 μm size fraction for the air-dried and glycolated specimens of the sediments from core EAP13-GC16B

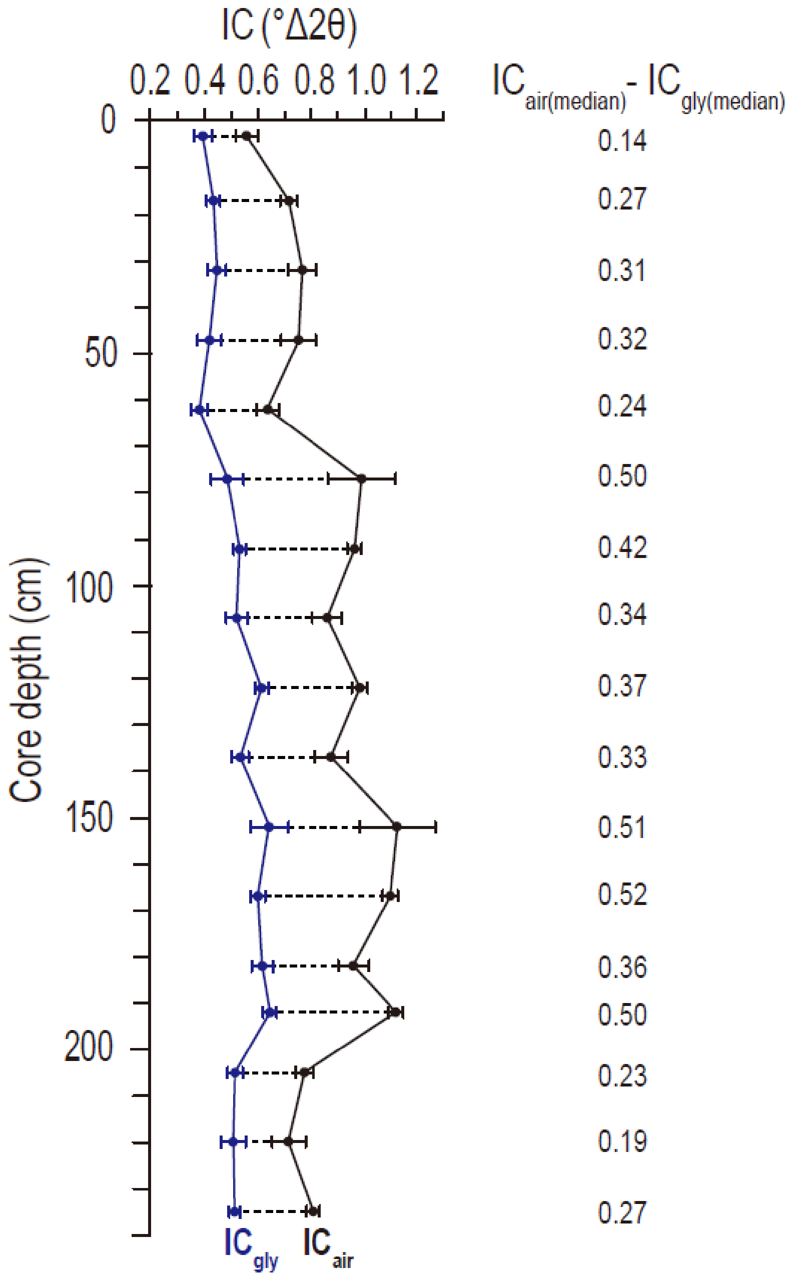

Supplementary Figure 3. Comparison of IC_{air} (air-dried samples) and IC_{gly} (ethylene glycolated samples) with increasing depth. Three independent measurements for each sample were made (see supplementary Table 2). Variation in the median values of IC with depths ($IC_{air\ (median)} - IC_{gly\ (median)}$) ranges 0.14-0.52 that corresponds to 5-8 % of smectite contents in illite/smectite mixed-layers.

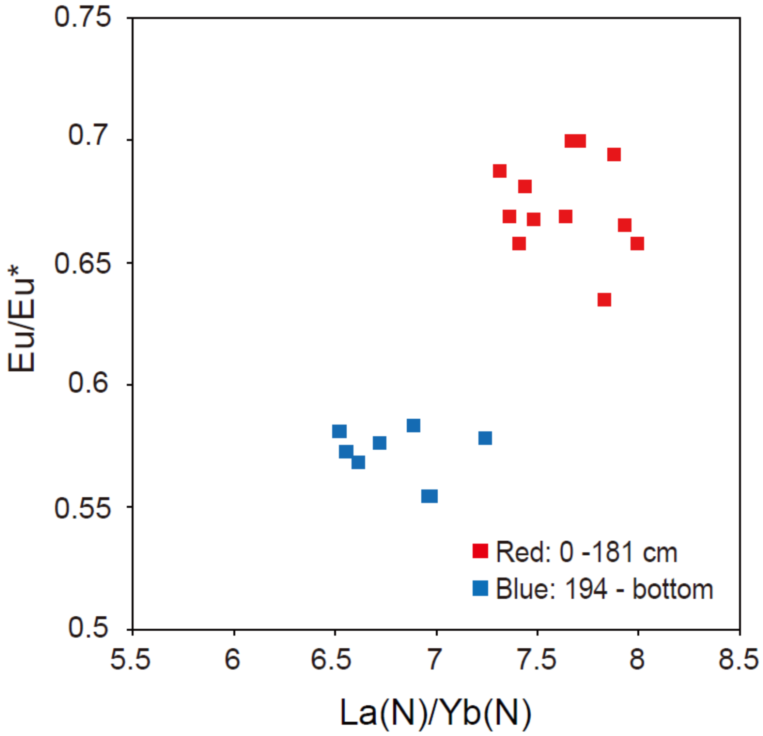

Supplementary Figure 4. Rare earth element compositions (Eu/Eu* and La(N)/Yb(N)) for bulk sediment from core EAP13-GC16B. Rare earth element composition indicates two different sources of illite from sediments of the LGM (U4) and the Holocene (U1-3) (Red: 0-181 cm, Blue: 194-bottom).

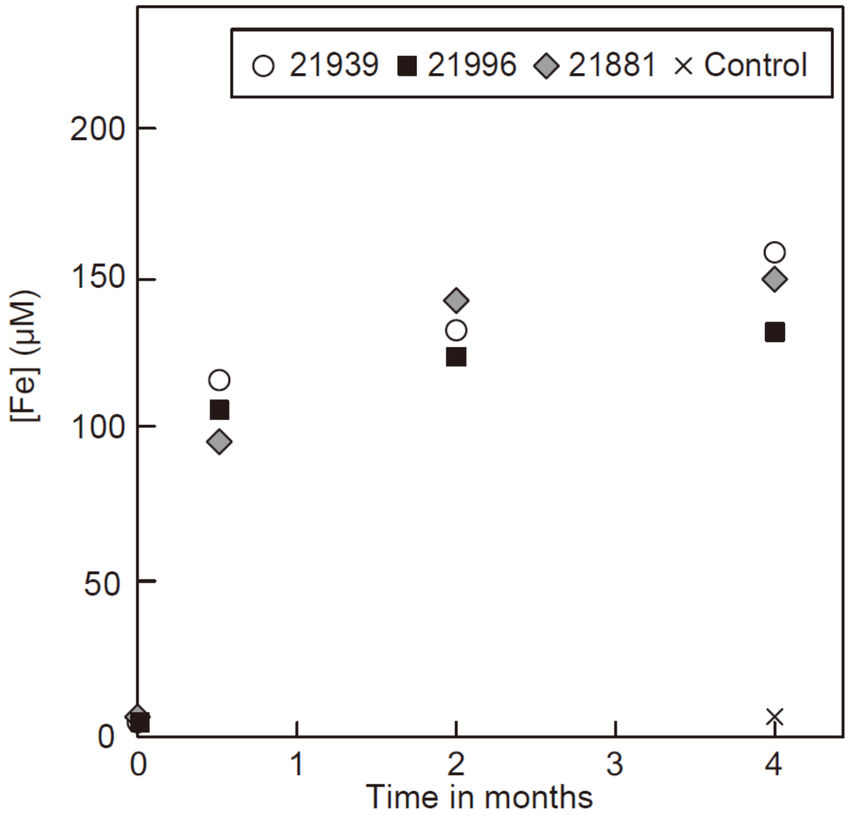

Supplementary Figure 5. Concentration of dissolved Fe in solution sample at various incubation times. Bioreduced and nonreduced control sample were incubated at 4 °C using psychrophilic bacteria (*Shewanella vesiculosa* 21939, 21996, and *Shewanella frigidimarina* 21881) isolated from the Antarctic Peninsula.
