# Supplementary material for: Microbial Fe(III) reduction as a potential iron source from Holocene sediments beneath Larsen Ice Shelf
Source: Nat Commun. 2019 Dec 19;10:5786. doi: 10.1038/s41467-019-13741-x (PMC6923428; doi:10.1038/s41467-019-13741-x)
Supplement: Supplementary file 3 — Reporting Summary [file 41467_2019_13741_MOESM3_ESM.pdf]

nature research

Corresponding author(s): DBPR, NCOMMS-19-02234B

Last updated by author(s): Nov 8, 2019

Reporting Summary

Nature Research wishes to improve the reproducibility of the work that we publish. This form provides structure for consistency and transparency in reporting. For further information on Nature Research policies, see Authors & Referees and the Editorial Policy Checklist.

Statistics

For all statistical analyses, confirm that the following items are present in the figure legend, table legend, main text, or Methods section.

n/a	Confirmed	
☐	☒	The exact sample size (*n*) for each experimental group/condition, given as a discrete number and unit of measurement
☒	☐	A statement on whether measurements were taken from distinct samples or whether the same sample was measured repeatedly
☒	☐	The statistical test(s) used AND whether they are one- or two-sided<br>*Only common tests should be described solely by name; describe more complex techniques in the Methods section.*
☒	☐	A description of all covariates tested
☐	☒	A description of any assumptions or corrections, such as tests of normality and adjustment for multiple comparisons
☐	☒	A full description of the statistical parameters including central tendency (e.g. means) or other basic estimates (e.g. regression coefficient) AND variation (e.g. standard deviation) or associated estimates of uncertainty (e.g. confidence intervals)
☐	☒	For null hypothesis testing, the test statistic (e.g. *F*, *t*, *r*) with confidence intervals, effect sizes, degrees of freedom and *P* value noted<br>*Give P values as exact values whenever suitable.*
☒	☐	For Bayesian analysis, information on the choice of priors and Markov chain Monte Carlo settings
☒	☐	For hierarchical and complex designs, identification of the appropriate level for tests and full reporting of outcomes
☒	☐	Estimates of effect sizes (e.g. Cohen's *d*, Pearson's *r*), indicating how they were calculated

Our web collection on statistics for biologists contains articles on many of the points above.

Software and code

Policy information about availability of computer code

Data collection	Gatan Inc.'s Digital Micrograph(DM) software was used to measured and analysed the TEM lattice fringe images. Search-Match software was used to measured the X-ray profiles of samples and identified the mineral assemblages.
Data analysis	OriginPro8 and Adobe Illustrator were used to plot the figures and tables. Microsoft Excel was used to calculate the mean and standard deviations.

For manuscripts utilizing custom algorithms or software that are central to the research but not yet described in published literature, software must be made available to editors/reviewers. We strongly encourage code deposition in a community repository (e.g. GitHub). See the Nature Research guidelines for submitting code & software for further information.

Data

Policy information about availability of data

All manuscripts must include a data availability statement. This statement should provide the following information, where applicable:

- Accession codes, unique identifiers, or web links for publicly available datasets
- A list of figures that have associated raw data
- A description of any restrictions on data availability

A list of figures that have associated raw data.

Field-specific reporting

Please select the one below that is the best fit for your research. If you are not sure, read the appropriate sections before making your selection.

☐ Life sciences ☐ Behavioural & social sciences ☒ Ecological, evolutionary & environmental sciences

Ecological, evolutionary & environmental sciences study design

All studies must disclose on these points even when the disclosure is negative.

Study description	Microbial alteration as a potential Fe source under the Larsen Ice Shelf C during the Holocene
Research sample	Continental shelf marine sediments
Sampling strategy	We used the gravity core to collect the marine sediments.
Data collection	Shipboard data collection as well as laboratory experiments
Timing and spatial scale	ANA03C Cruise Expedition by the Korea Polar Research Institute is conducted from 4th April to 15th May to collect the sediments samples.
Data exclusions	No data was excluded.
Reproducibility	3sets of IC data were measured from the independent XRD data. We showed the SD for each data point.
Randomization	The authors are involved in the Antarctic research program operated by KOPRI. Previously, authors worked together before, so that we know the expertise from each author. Corresponding author controlled the sample allocation.
Blinding	Our shipboard core property measurement or XRD measurement does not require the blinding. These are direct measurement by the instruments. XRD analysis for IC was also performed by Chebyshev polynomial with ≤20 coefficients, and the pseudo-Voigt profile function. Therefore, there is no blinding is required.

Did the study involve field work? ☒ Yes ☐ No

Field work, collection and transport

Field conditions	Low temperature, windy and lots of sea-ice
Location	Northwestern part of Larsen Ice Shelf-C embayment (66º 3.898' S, 60º 27.692' W, 324 m depth), Antarctica
Access and import/export	Ice breaker R/V Araon
Disturbance	No disturbance.

Reporting for specific materials, systems and methods

We require information from authors about some types of materials, experimental systems and methods used in many studies. Here, indicate whether each material, system or method listed is relevant to your study. If you are not sure if a list item applies to your research, read the appropriate section before selecting a response.

Materials & experimental systems

n/a	Involved in the study	
☒	☐	Antibodies
☒	☐	Eukaryotic cell lines
☒	☐	Palaeontology
☒	☐	Animals and other organisms
☒	☐	Human research participants
☒	☐	Clinical data

Methods

n/a	Involved in the study	
☒	☐	ChIP-seq
☒	☐	Flow cytometry
☒	☐	MRI-based neuroimaging
